# MUVY: A multi-view user-generated sports video dataset

**DOI:** 10.1016/j.dib.2026.113003

**Published:** 2026-06-20

**Authors:** Larissa Pessoa, Elton Alencar, Rosiane de Freitas

**Affiliations:** Institute of Computing, Federal University of Amazonas, *Av*. Gen. Rodrigo Octávio, 6200 Setor Norte do Campus Universitário - Coroado, Manaus, AM, Brazil

**Keywords:** Camera position annotation, Event recognition, Multi-view videos, Object detection, Sports video analysis, User-generated content, Video understanding

## Abstract

The MUVY dataset contains user-generated video recordings of real-world sporting events captured from multiple viewpoints using handheld mobile devices within sports venues. The dataset was constructed from publicly available Creative Commons-licensed YouTube videos and organized to preserve the multi-view relationships between independently recorded camera perspectives of the same sporting action. MUVY comprises 141 video recordings grouped into 31 events across seven sports categories, including athletics, soccer, basketball, American football, artistic gymnastics, tennis, and cricket, totaling approximately 3 h and 15 min of footage. Each recording is accompanied by video-level metadata and frame-level spatial annotations, including manually assigned camera viewpoint labels and object detection annotations identifying players, referees, goalkeepers, and sports ball classes. The dataset is organized in a hierarchical structure by sport category, event identifier, and camera recording, providing access to the original videos, extracted image frames, and associated annotation files. MUVY may support research on multi-view video analysis tasks that involve spatial reasoning and viewpoint-aware processing in unconstrained acquisition conditions.

Specifications Table

**Table:** 

Subject	Computer Science, Computer Vision and Video Understanding
Specific subject area	Multi-view video understanding from user-generated sports recordings
Type of data	Video (.mp4), Image frames (.jpg), Metadata files (.txt)
Data collection	Videos were manually selected from Creative Commons-licensed YouTube recordings capturing the same sporting event from different viewpoints. Python scripts were used to extract the video content, which includes the recording file (.mp4), metadata (.txt), and image frames (.jpg). Metadata, such as the video identifier, title, duration, and source URL, were automatically extracted. Camera positions were manually annotated, and object detection annotations were generated using YOLO-World and manually validated.
Data source location	YouTube (Creative Commons licensed videos)
Data accessibility	Data identification number DOI: https://doi.org/10.5281/zenodo.13883315 Direct URL to data: https://zenodo.org/records/13883315 Additional information regarding the dataset structure, organization, examples, and access instructions is available through the MUVY project webpage: https://swperfi-project.github.io/Pages-dev/MuvyDataset-page/ https://github.com/swperfi-project/muvy-dataset-scripts The original YouTube URLs are maintained in the metadata files for reference purposes. To improve long-term accessibility independently of the availability of the original online videos, the dataset repository provides extracted image frames, metadata files, and annotation files for all 141 recordings included in the dataset. The complete dataset occupies approximately 7.2 GB of storage, including extracted frames, metadata files, and annotation files. The dataset can be accessed using standard video processing and computer vision libraries capable of handling JPG and TXT file formats. A minimal replication pipeline, including frame extraction and YOLO-World inference scripts, is publicly available through the project repository.
Related Research Article	PESSOA, Larissa et al. Exploring multi-camera views from user-generated sports videos. In: Symposium on Knowledge Discovery, Mining and Learning (KDMiLe). SBC, 2024. p. 105–112.

## 1 Value of the Data

• The MUVY dataset provides multi-view recordings of real-world sporting events captured from independently operated handheld mobile devices positioned at different locations within sports venues.
• The dataset contains recordings associated with the same sporting actions or event moments observed from multiple viewpoints, enabling the study of cross-view relationships across independently captured video streams.
• In addition to the video recordings, the dataset provides recording-level metadata, manually assigned camera viewpoint labels, and frame-level object detection annotations, enabling the association of spatial observations across different viewpoints.
• Because the recordings originate from unconstrained user-generated videos, the dataset includes natural variations in camera motion, framing, occlusion, viewpoint positioning, zoom, and recording quality commonly observed in spectator-generated content.
• Recordings associated with the same sporting moments and captured from independently recorded viewpoints may support studies involving temporal alignment, replay grounding, and cross-view player association across distinct camera perspectives.
• The combination of frame-level spatial annotations, manually assigned viewpoint labels, and unconstrained handheld recordings may support multi-object tracking, cross-view object re-identification, viewpoint classification, viewpoint-aware retrieval, camera pose estimation, camera selection strategies, spectator-sourced video fusion, multi-view representation learning, self-supervised or contrastive pretraining with multi-view pairs, video mashup generation, and camera calibration-free fusion approaches.

## 2 Background

The widespread use of mobile devices equipped with video recording capabilities has contributed to the growth of user-generated audiovisual content shared through online platforms [1,8]. Public video platforms such as YouTube have also become sources of reusable video data for computer vision benchmarks and related video understanding tasks [1]. In parallel, multi-camera and multi-view systems have been used to support scene understanding by combining observations captured from different viewpoints [9]. In sports contexts, recordings acquired from multiple positions inside a venue can provide complementary visual information that is not observable from a single camera perspective, which is relevant for tasks involving event understanding, camera viewpoint analysis, spatial localization, and re-identification [[4], [5], [6]].

Existing datasets for sports video understanding, such as SoccerNet and SoccerNet-v2, have supported progress in tasks including action spotting and holistic understanding of broadcast soccer videos [5,6]. Additional sports datasets, such as MultiSports, provide spatio-temporally localized sports actions at scale [7]. However, these resources are typically based on broadcast footage or are not specifically organized around independently recorded user-generated viewpoints of the same event [[5], [6], [7]]. Related data articles have also described video datasets with frame-level annotations and in-the-wild acquisition conditions, reinforcing the relevance of structured video resources for computer vision reuse [13,14].

Other multi-view datasets illustrate the relevance of unconstrained or mobile acquisition, including the Multi-Viewpoint Outdoor Action Recognition dataset, the Multi-sensor Concert Recording Dataset, and the Jiku Mobile Video Dataset [2,10,11]. These datasets are useful references for multi-view analysis, but they target different domains or acquisition settings than user-generated sports recordings captured simultaneously by spectators during live events [2,10,11]. To support the study of multi-view relationships in user-generated sports recordings captured under unconstrained conditions, the MUVY dataset was compiled from publicly available videos capturing the same sporting events from different viewpoints. Unlike broadcast-based datasets, the recordings originate from handheld spectator devices, reflecting realistic acquisition conditions commonly observed in user-generated sports videos. An earlier version of this dataset and a preliminary analysis of multi-view relationships were presented in [12]. The earlier version introduced in [12] contained a smaller collection of sporting events and recordings focused on preliminary multi-view analysis. The current version expands the dataset to 141 recordings distributed across 31 sporting events and seven sports categories, together with additional metadata organization and frame-level object detection annotations. In addition, the present manuscript provides a more detailed description of the dataset construction process, annotation procedures, and hierarchical organization structure.

## 3 Data Description

The MUVY dataset contains user-generated videos capturing real-world sporting events from multiple viewpoints. The recordings are organized to preserve the multi-view relationships within each event. The dataset contains 141 videos grouped into 31 distinct events across seven sports categories: athletics, soccer, basketball, American football, artistic gymnastics, tennis, and cricket (see Table 1).

**Table 1:** Dataset composition by sport category.

Sport Genre	Events	Videos	Frames	Duration
Athletics	8	50	174,388	1:46:33
Soccer	8	35	70,774	0:39:48
Basketball	5	13	14,105	0:07:53
American Football	4	30	44,692	0:24:55
Artistic Gymnastics	3	6	18,841	0:10:28
Tennis	2	5	7064	0:03:55
Cricket	1	2	3316	0:01:51
**TOTAL**	**31**	**141**	**333,180**	**3:15:23**

**Fig. 1:**
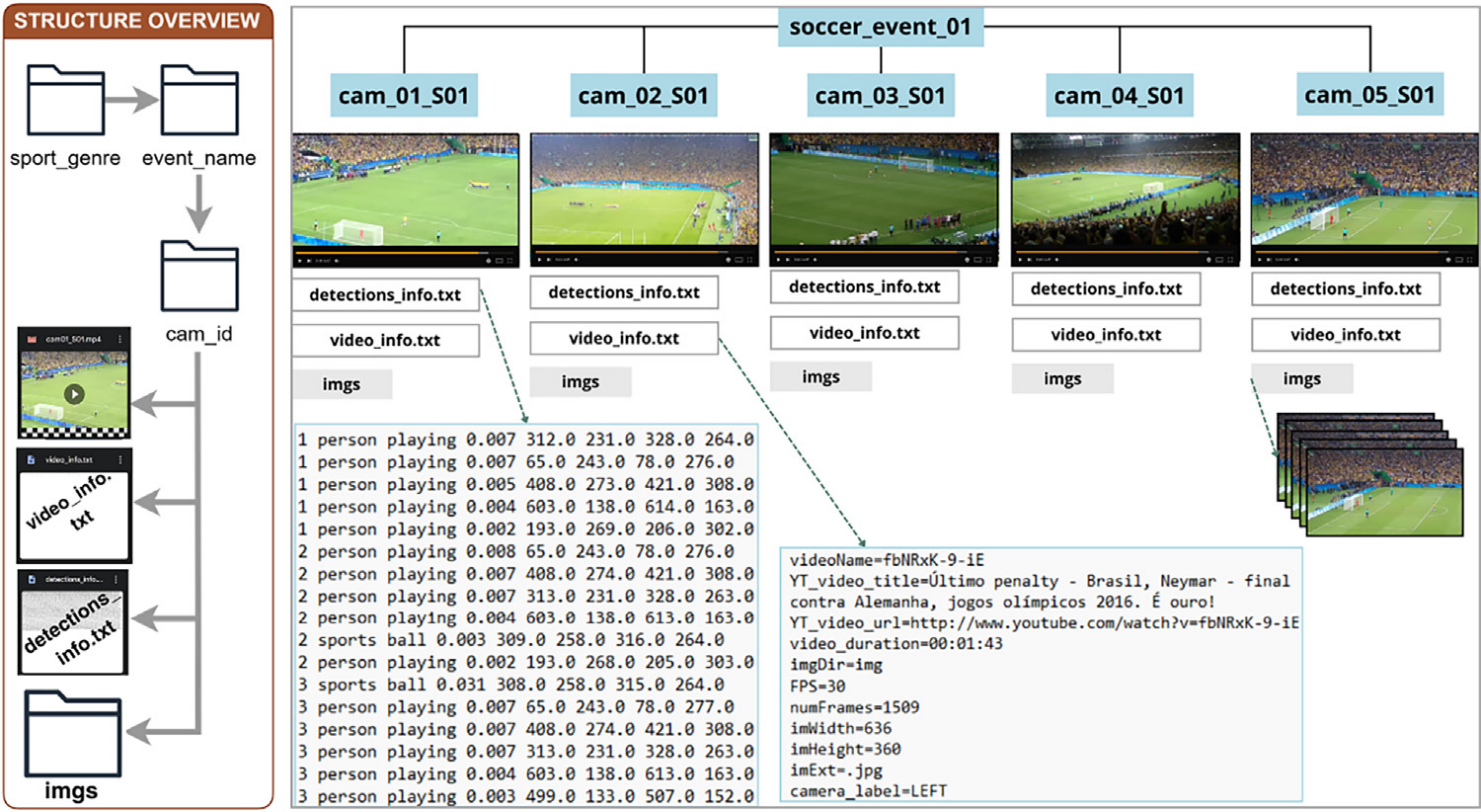
An example of a soccer event with five different cameras. Each camera includes a video from its viewpoint, a file with annotations of detected objects, a file with video metadata, and a directory with extracted frames.

The total duration of the dataset is approximately 3 h and 15 min, corresponding to 333,180 extracted frames. Videos are organized into a hierarchical directory structure according to sport category and event identifier. As illustrated in Fig. 1, each event directory contains multiple recordings corresponding to different viewpoints of the same action. Table 1 reports the dataset composition by sport category.

The dataset contains an average of approximately 4.5 recordings per event, with the number of viewpoints per event ranging from 2 to 17 recordings. Athletics recordings correspond to the largest portion of the dataset in terms of number of videos and extracted frames. Videos are organized in a hierarchical directory structure by sport category and event identifier, where each event directory contains multiple camera recordings corresponding to different viewpoints of the same scene. Each recording is stored in a dedicated directory associated with a specific camera perspective. Within each camera directory, the dataset provides: a) the original video file in *MP4* format; b) a metadata file named *video_info.txt*; c) a frame-level object detection annotation file named *detections_info.txt*; d) and a folder containing the extracted image frames in *JPG* format (img/).

An example of the dataset structure for a single event is illustrated in Fig. 1, showing the hierarchical organization adopted in MUVY, where each event directory contains multiple camera recording subdirectories associated with different viewpoints of the same sporting moment. Each camera directory includes the corresponding video recording, metadata file, object detection annotation file, and extracted image frames. This organization preserves the correspondence between recordings, metadata, extracted frames, and annotation files at the event level.

The representation of multi-perspective observations of real-world sports scenarios is enabled by the described structure where each event in the dataset contains multiple video recordings capturing the same sporting action from distinct viewpoints within the sports venue. This arrangement also facilitates the retrieval and comparison of corresponding observations across different camera perspectives.

For each camera recording, the dataset provides a metadata file named video_info.txt, which stores video-level information including the event identifier, *YouTube* video identifier and *URL*, video duration, frame rate (*FPS*), number of extracted frames, spatial resolution, image file extension, and the assigned camera viewpoint label. The dataset contains recordings with varying spatial resolutions inherited from the original user-generated videos available on YouTube. Most videos are concentrated in two common resolutions, 1280 × 720 pixels (60 videos) and 640 × 360 pixels (58 videos), while the remaining recordings appear in less frequent resolutions such as 480 × 360, 360 × 640, 636 × 360, 270 × 480, 638 × 360, 360 × 360, 568 × 320, 304 × 360, and 406 × 720 pixels. Considering the total pixel area, the resolution range extends from 304 × 360 pixels to 1280 × 720 pixels in terms of total pixel area. Camera viewpoints are categorized according to their relative position with respect to the sports field into five predefined classes: LEFT, BEHIND_LEFT, CENTER, RIGHT, and BEHIND_RIGHT, see Fig. 3.

For sports with non-rectangular or less standardized spatial layouts, the viewpoint labels were assigned according to the relative position of the camera with respect to the primary activity area observed in the recording. In athletics, the CENTER label was used when the camera was approximately aligned with the main track segment or action area being observed, while LEFT and RIGHT were assigned according to the direction of the race or movement in that segment. In artistic gymnastics, the reference area was the apparatus being recorded; when no specific apparatus was present, the central performance area was used as the spatial reference. For basketball, the same relative field/court-based interpretation adopted for rectangular sports such as soccer was applied. The predominance of CENTER labels in athletics reflects the natural distribution of the available user-generated recordings rather than an imposed balancing decision.

Table 2 presents the distribution of camera viewpoint labels across sports categories. Athletics recordings correspond predominantly to the CENTER viewpoint category, while sports such as soccer and American football present a broader distribution of lateral and behind-field viewpoints.

**Table 2:** Distribution of camera viewpoint labels across sports categories.

Sport Genre	LEFT	BEHIND_LEFT	CENTER	RIGHT	BEHIND_RIGHT	TOTAL RECORDINGS
American Football	17.2%	3.4%	6.9%	58.6%	13.8%	**30**
Artistic Gymnastics	16.7%	0.0%	33.3%	50.0%	0.0%	**6**
Athletics	4.0%	0.0%	88.0%	8.0%	0.0%	**50**
Basketball	38.5%	15.4%	7.7%	15.4%	23.1%	**13**
Cricket	0.0%	0.0%	100.0%	0.0%	0.0%	**2**
Soccer	17.1%	11.4%	8.6%	37.1%	25.7%	**35**
Tennis	20.0%	0.0%	20.0%	60.0%	0.0%	**5**

These viewpoint labels describe the spatial placement of each recording within the event environment and enable the association of corresponding multi-view observations across different camera perspectives. Recordings associated with the same event were manually matched through visual inspection to ensure that they corresponded to the same sporting moment, such as a penalty kick, goal, play, or competition action, observed from different viewpoints. The matching process considered the observed action, athlete positioning, scene progression, and event context across recordings. However, the dataset does not provide frame-level or timestamp-level synchronization between viewpoints. Therefore, while recordings correspond semantically to the same sporting moment, precise temporal alignment across views may require additional processing depending on the target application.

Frame-level object detection annotations are also provided for each recording in the associated *detections_info.txt* file. In total, the dataset contains 208,111 frame-level object detection annotations, corresponding to an average of 3.14 detections per annotated frame. Each annotation entry corresponds to a detected object within a specific frame and includes the object class label and bounding box coordinates expressed in pixel units which indicate the object spatial position. The structure of the detection annotation records provided in the detections_info.txt files is described in Table 3.

**Table 3:** Detection annotation record format provided in the *detections_info.txt* files.

Field	Data type	Description
frame_id	Integer	Identifier of the extracted frame
detect_id	Integer	Detection identifier
object_class_name	String	Detected object category
x1	Float	Bounding box left coordinate
y1	Float	Bounding box top coordinate
x2	Float	Bounding box right coordinate
y2	Float	Bounding box bottom coordinate
confidence_score	Float	YOLO-World detection confidence score

## 4 Experimental Design, Materials and Methods

**Fig. 2:**
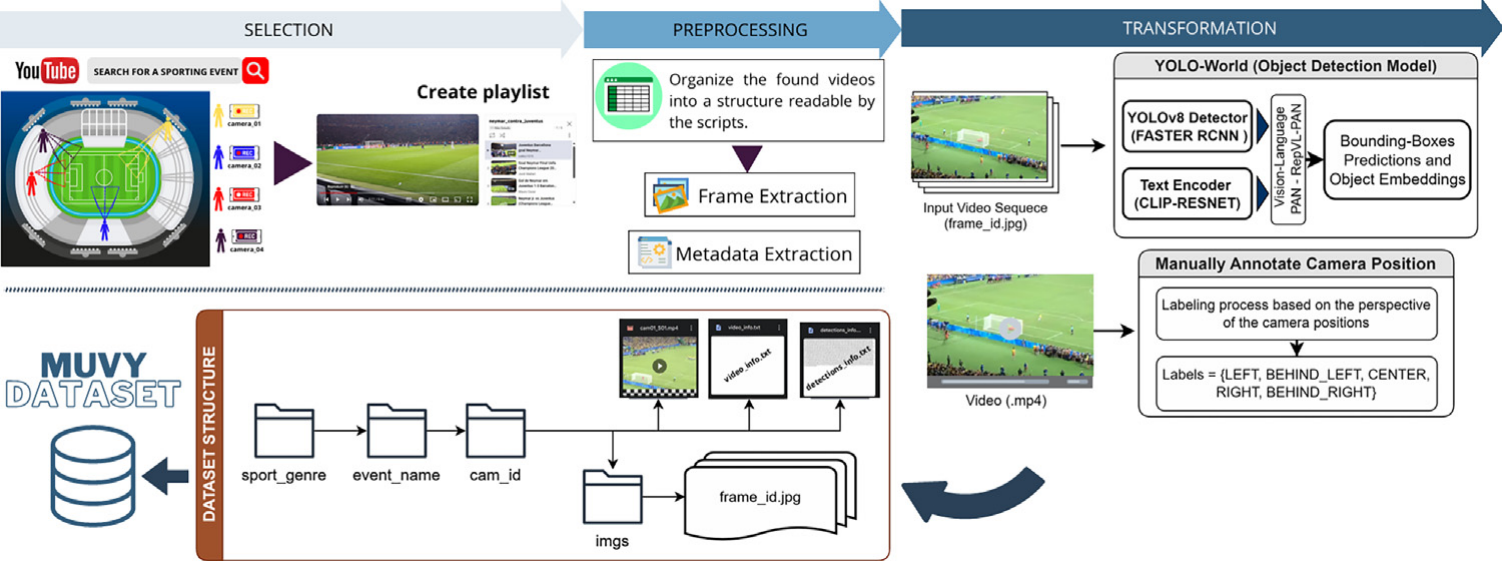
Dataset construction pipeline: searching, collecting, and structuring the extracted sports video content. Adapted and expanded from [12].

This section describes the workflow implemented to construct the MUVY dataset from publicly available multi-view sports videos. The construction process comprises four stages: (i) video acquisition and event grouping, (ii) preprocessing and content extraction, (iii) annotation generation, and (iv) final dataset structuring. An overview of the dataset construction pipeline is presented in Fig. 2.

### 4.1 Multi-view video acquisition and event grouping

Multi-view recordings included in the MUVY dataset were identified from publicly available videos on YouTube, restricted to content distributed under a Creative Commons license. The search process was manually conducted using keyword-based queries composed of three elements: (i) sport genre, (ii) a textual description of an in-game action (*e.g., goal, penalty, mistake*), and (iii) competition season or tournament name (*e.g., World Cup, Olympic Games*). The event selection procedure also included the exclusion of duplicated uploads, edited highlight compilations, recordings with severe visual corruption, and videos that did not provide sufficient overlap with other viewpoints of the same sporting moment. A complete list of the grouped events and their corresponding source videos is provided through the project webpage associated with the dataset.

The objective of this stage was to identify sets of videos capturing the same sporting moment simultaneously from different positions inside the venue, allowing the combination of multi-view observations of a single event. To ensure this property, search queries were iteratively refined until at least two recordings capturing the same sporting action from distinct viewpoints could be identified for a given event (*e.g., “Final Penalty Goal – Rio 2016 Olympic Final Brazil vs. Germany”*).

Once identified, recordings corresponding to the same sporting event/action were grouped into multi-view sets using YouTube playlists. Each playlist represents a single sporting event and contains two or more videos that capture the same action concurrently from different viewpoints. These playlists were subsequently used as the input source for the automated acquisition and preprocessing pipeline described in Section 2.

Videos were included in the dataset if they: (i) were publicly available under a Creative Commons license on YouTube, (ii) contained recordings of identifiable sporting actions or event moments, and (iii) could be associated through manual inspection with at least one additional recording capturing the same sporting moment from a different viewpoint, considering visual correspondence between sporting actions, athlete positioning, scene progression, and event context.

### 4.2 Preprocessing and content extraction

Following the grouping of multi-view recordings into event-specific YouTube playlists, the corresponding video URLs were exported into a structured CSV file to support manual verification and adjustment prior to automated processing. This intermediate step enabled the correction of inaccessible links, removal of duplicate entries, and standardization of playlist records for downstream processing. A Python-based processing pipeline was implemented to extract the content related to the selected recordings and extract basic video-level metadata, including the YouTube video identifier, title, URL, and duration.

Frames were extracted using the native frame rate of each recording in order to preserve the original temporal characteristics of the videos. Most recordings were captured at 30 FPS, while a smaller subset was available at 25 FPS. The number of extracted image files in the corresponding *img/* directory matches the *numFrames* value reported in *video_info.txt*, indicating that all frames were extracted for each recording. No additional frame resizing or filtering procedures were applied during frame extraction. These frame sequences were stored in JPEG format in a directory associated with each recording. In addition, a recording-level metadata file (*video_info.txt*) was produced to store information required for indexing and downstream usage (*e.g., frame rate, spatial resolution, and number of extracted frames).* The outputs produced in this stage were subsequently used as inputs for the annotation procedures described in Section 3.

### 4.3 Annotation procedure

To enrich the dataset with spatial and viewpoint-related information, two complementary annotation procedures were applied to each recording: (i) camera viewpoint annotation and (ii) frame-level object detection annotation.

#### 4.3.1 Camera viewpoint annotation

**Fig. 3:**
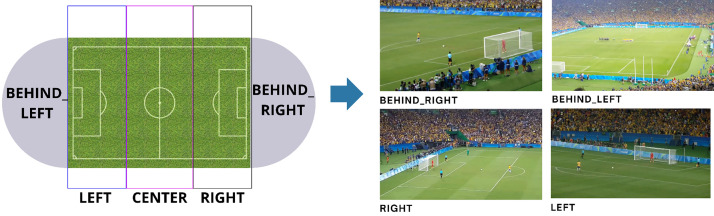
Labeling of camera perspectives. The top-left diagram shows the layout of a rectangular sports field divided into five distinct camera positions. The accompanying images display examples of views from each labeled camera position during a soccer match. Adapted and expanded from [12].

Each recording was manually assigned a camera viewpoint label describing the relative position of the camera with respect to the sports field. As illustrated in Fig. 3, the viewpoint categorization follows five discrete classes: *LEFT, BEHIND_LEFT, CENTER, RIGHT, and BEHIND_RIGHT*.

These labels were defined based on commonly observed camera placement configurations in sports recorded within rectangular playing areas, such as soccer and tennis. The viewpoint labels were assigned by three manual annotators through case-by-case visual inspection. For each event, annotators examined both the individual recording and the set of videos associated with the same sporting moment in order to understand the complete scene context and select the label that best represented the camera position. This procedure was particularly important for sports with non-rectangular layouts or event-specific spatial references, where the label semantics were defined relative to the main action area rather than strictly to a fixed rectangular field geometry. The assigned viewpoint information is stored at the recording level in the *video_info.txt* file associated with each camera directory.

#### 4.3.2 Object detection annotation

Frame-level object detection annotations were generated for the extracted image frames using an automated detection procedure based on the YOLO-World model [3] with its pre-trained open-vocabulary object detection configuration. The object classes player, goalkeeper, referee, and sports ball were used as textual prompts during the detection procedure. YOLO-World/L was executed using a confidence threshold of 0.005 and *Non-Maximum Suppression* with an IoU threshold of 0.45 before manual validation. A low confidence threshold was adopted because open-vocabulary detections for sport-specific concepts may produce lower confidence scores than standard closed-set detections. The confidence scores produced during inference were retained in the final detections_info.txt files together with the object class labels and bounding box coordinates. The resulting detections were subsequently subjected to manual validation by the authors through visual inspection of the recordings in order to remove evident incorrect or inconsistent predictions. The validation procedure focused on identifying evident false detections, incorrect object class assignments, and inconsistent bounding box associations across the extracted frames. A formal inter-annotator agreement measurement was not computed, and correction/rejection rates were not recorded during dataset construction.

**Fig. 4:**
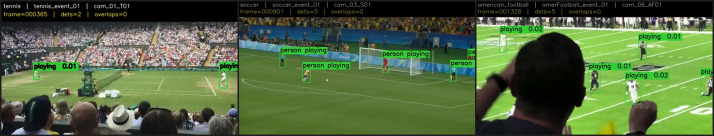
Example of frame-level object detection annotations in MUVY. Bounding boxes and class labels associated with detected objects are overlaid on extracted video frames from different sporting events.

The object detection annotations include classes of interest in sports scenarios, namely *player, goalkeeper, referee*, and *sports ball*. Each annotation entry corresponds to a detected object associated with a specific frame identifier and contains the predicted object class label, and the bounding box coordinates expressed in pixel units. The bounding box coordinates are represented by the values (*x1, y1, x2, y2*), corresponding to the top-left and bottom-right corners of the detected region within the extracted frame. An example of the generated object detection annotations is shown in Fig. 4, illustrating detected objects associated with different frames across multiple sporting events and camera viewpoints.

Detection entries are stored sequentially in the *detections_info.txt* file associated with each recording, preserving the correspondence between frame identifiers and detected spatial regions across the frame sequence.

### 4.4 Final dataset structuring

The outputs generated during the acquisition, preprocessing, and annotation stages were consolidated into a hierarchical directory structure designed to preserve the multi-view relationships between recordings of the same sporting event. Collected video files, extracted frame sequences, recording-level metadata, and frame-level annotation files were systematically organized according to sport category, event identifier, and camera recording. This organization ensures that each recording maintains an explicit association between the original video content, its extracted frames, the corresponding metadata stored in *video_info.txt*, and the object detection annotations stored in *detections_info.txt*. The resulting dataset structure enables the retrieval of multi-view observations of the same sporting action while preserving the spatial and viewpoint-related annotations generated during the previous stages.

## Limitations

The MUVY dataset is constructed from publicly available Creative Commons-licensed recordings, and its composition depends on the availability of user-generated videos capturing the same sporting event from multiple viewpoints. Consequently, the number of recordings and viewpoints per event varies across sports categories, and the distribution of recordings and extracted frames across sports categories is not uniform. As a result, downstream learning-based tasks may be influenced by class imbalance across sports categories and viewpoint distributions. This imbalance may affect the generalizability of models trained directly on MUVY, especially when the objective is to learn representations that perform uniformly across different sports categories or viewpoint classes. Models may be biased toward the visual patterns of more represented sports, such as athletics and soccer, or toward more frequent camera viewpoints, such as CENTER in athletics. Therefore, depending on the target task, users may consider sport-stratified and/or viewpoint-stratified sampling strategies during training, validation, and testing.

However, the dataset organization was designed to facilitate the incorporation of additional recordings and events if future extensions of MUVY are developed. Any future updates may be documented through the project webpage and associated repository records to support traceability across dataset versions. The spatial distribution of camera viewpoints within each event is not uniform and reflects the positions of spectators during live matches. Since the dataset is based on publicly available user-generated recordings, the distribution of sports categories, camera viewpoints, recording devices, and event contexts depends on the availability of online content and spectator recording behavior. In addition, although recordings associated with the same event were visually matched to represent the same sporting moment, such as a penalty kick, goal, play, or competition action, the dataset does not provide frame-level or timestamp-level synchronization across viewpoints. Consequently, some events may contain a higher concentration of recordings from specific viewing angles, while other viewpoints may be underrepresented. Object detection annotations focus on a predefined set of classes relevant to sports analysis scenarios, namely player, goalkeeper, referee, and sports ball. These categories were selected to support tasks related to player localization, game dynamics analysis, and multi-view object association. Consequently, other objects that may appear in the scene are not annotated.

## Ethics Statement

The authors of this work declare that the paper meets the journal's requirements, guaranteeing that it is original and has not been submitted to any other venue. The research does not involve animal experiments or private/restricted user data. The dataset was constructed from publicly available YouTube videos identified through the platform's Creative Commons license filter. The videos were downloaded for dataset construction, and their original YouTube URLs were preserved in the metadata files for source traceability. The use and redistribution of the dataset should be interpreted in accordance with the applicable Creative Commons license terms and YouTube's Terms of Service.

## CrediT Author Statement

**Larissa Pessoa, Elton Alencar:** Conceptualization, Methodology, Data curation, Implementation, Writing – Original draft preparation, Investigation; **Rosiane de Freitas**: Conceptualization, Methodology, Project administration, Supervision, Writing – Reviewing and Editing.

## Data Availability

ZenodoMUVY: A multi-view user-generated sports video dataset (Original data).
